# Understanding definitions of visual impairment and functional vision

**Published:** 2020-12-31

**Authors:** Vaishali KV, P Vijayalakshmi

**Affiliations:** 1 Fellow in paediatric ophthalmology and strabismus: Aravind eye hospital, Madurai, India.; 2 Professor of Ophthalmology and Chief, Department of paediatric ophthalmology and adult strabismus: Aravind eye hospital, Madurai, India.

**The legal definition of blindness assumes great importance in extending device-related support to those suffering from vision-related problems. Whereas Visual Acuity (VA) alone does not determine the quality of vision, certain other factors too have to be considered, specifically for the issue of driving licenses.**

**Figure:**
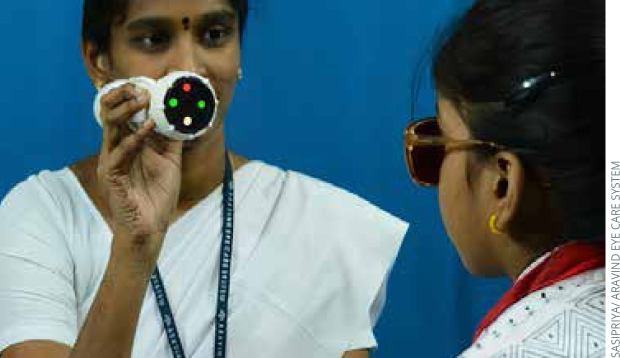
Assessment of binocularity using Worth's four dot test.

Globally, 253 million people are estimated to be visually impaired, of whom 36 million are blind. In the recent past, significant changes were made to the definitions of visual impairment and blindness, which has ensured that vision loss due to uncorrected refractive error is not excluded when estimating the burden of visual impairment.

The World Health Organisation has adopted the International Classification of Diseases 11 (ICD 11) definition of visual impairment and blindness. According to this definition, a person is said to be visually impaired if the presenting VA in the better eye is worse than 3/60. In this revised definition, near vision impairment is also included; it is defined as presenting near VA worse than N6 with existing correction.

Decision-makers in government use definitions of blindness and vision impairment when formulating policies to provide financial support and other benefits for those certified to be legally blind. For instance, in India, people who are legally blind can avail free public transportation and the assistance of scribe during examinations, etc.

The definition of blindness under India's National Programme for Control of Blindness (NPCB) is different from the current definition adopted by the World Health Organization in the International Classification of Diseases - 10 (ICD-10). The VA criteria under which an individual will be classified as blind in the two definitions are different, being 20/200 in the NPCB definition, and 20/400 in the ICD-10 definition after the 2006 revision. In India, the Ministry of Social Justice and Empowerment (MoSJE) is responsible for developing programmes that extend support to people with disabilities. As a recent initiative, the ministry has recommended that the revision of the blindness be cut off to 20/400 instead of 20/200 that is currently followed by NPCB. The only difference is the MoSJE classification will be applied to any person based on the best-corrected VA, and not the presenting VA. It is essential that the same criteria be used universally under all the programs associated with policymaking and service provision for the blind and the visually impaired.

Another important definition pertains to functional low vision. This is defined as ‘VA less than 6/18 up to the perception of light, with treatment and best possible refractive correction, or visual field less than 10 degrees from the point of fixation’. This is an important definition for the sake of the evaluation, because a person with this vision may benefit from low-vision devices and services. Such a person must be encouraged and trained to maximise the potential benefit from the residual vision, often with the help of assistive devices and specific lifestyle changes.

It is important to note that the VA alone does not determine the overall quality of vision required for optimal functioning. The functional assessment of the vision comprises the visual field, colour vision, stereopsis, extraocular motility, contrast sensitivity, glare sensitivity and night vision, besides VA. All these are considered for specific critical licensing procedures, such as the issue of driver's and pilot licences, and determining eligibility for civil services, etc.

Driving is a complex activity where the driver should consider multiple objects in the visual field, scan the moving objects with precision to exercise sound judgment, and recognise coloured signals. Thus, aspects such as visual field, colour vision and contrast sensitivity are considered necessary while issuing a driving licence in certain countries. In India, the issue of a driving license for light motor vehicles is based on self-assessment and issue of a medical certificate, that cover the following vision-related aspects:

- In case of presence of any refractive error, whether it has been corrected with suitable spectacles
- Visual acuity: The ability to read a vehicle's number plate from a distance of 25 metres, translates to a VA of 6/12, as the standard size of these characters is 65mm
- Colour vision: the ability to distinguish the primary colours - red and green
- Whether a person suffers from night blindness.

**Figure:**
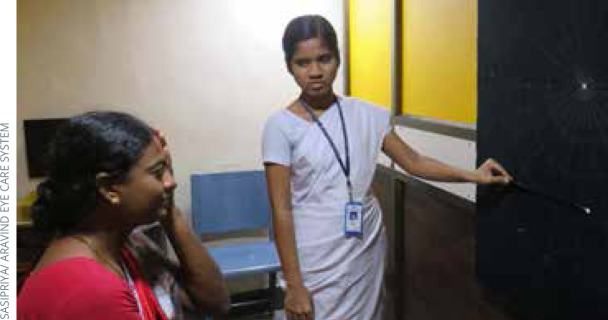
Assessment of central fields using Bjerrum screen. **INDIA**

In India, driving license is issued by Regional Transport Offices (RTO) in every state, and the procedure is regulated by the Motor Vehicle Act (MVA) 1988, amended in 2017.

For an unrestricted (non-commercial) driving licence, it is recommended that, with both eyes, the applicant should have a VA of 6/12 or better, and an uninterrupted visual field of 120° or better in the horizontal meridian. There are gaps in the mandatory standards for issuing driving licences, which need to be addressed.

According to the International College of Ophthalmologists' (ICO) visual standards for driving, regular testing of contrast sensitivity and glare sensitivity has to be done. The licences should be renewed after a definite period, and people above 65 years of age should be evaluated more frequently. The licences can be issued with restrictions if the need arises. For a commercial driving license, a VA of 6/9 in the better eye, and 140° horizontal field of vision is required. If the uncorrected VA is < 6/24, the application for the licence is rejected.

Monocular or one-eyed persons are eligible for the driver's license, if the VA in the remaining eye is 6/12 or better, the horizontal visual field is 120 degrees or more, and at least six months should have passed after the loss of vision in the eye, for monocular adaptation.

Extrapolating the results of a clinical examination and aiding in rehabilitation can be complemented by the functional assessment of vision. The functional vision assessment includes a variety of evaluations to understand the impact of visual impairment in the daily activities of life and guide them to make successful adaptations.

## Visual acuity

It is the spatial resolving capacity of the visual system and measures the sharpness of vision. Visual acuity is usually measured using a Snellen's chart at a distance of 6 metres or 20 feet. Several other charts are available for specialised conditions. Normal vision is denoted as a visual acuity of 6/6 or 20/20. Near VA measures the ability of a person to see objects at a working distance - usually about 40 cm or 16 inches.

## Visual fields

A visual field refers to the entire area that can be seen by the eye when fixated at a point. It is measured by a confrontation test, tangent screen, Humphrey's field analyser or Goldman perimetry. It is significant to note that the presence of normal visual fields enables orientation mobility and helps while searching. In the case of glaucoma, retinitis pigmentosa, and many other neurological conditions, there is a possibility of the presence of a defect in the visual fields. This can be in the form of central scotomas and peripheral constrictions. An Amsler's grid can be used to measure the central fields, as it is essential in ascertaining legal blindness.

## Colour vision

It is important to be able to distinguish colours well. The colour vision is assessed using D-15 panel test, and Ishihara pseudoisochromatic coloured plates. As part of rehabilitation, patients can be advised to seek high colour and tone contrast. The Ministry of Road Transport and Highways (MoRTH) amended Form 1 and Form 1A of the Central Motor Vehicles (CMV) Rules, 1989, to enable the citizens with mild to medium colour blindness, to obtain a driving licence. However, restrictions on severe colour blind citizens still apply, with respect to driving.

## Contrast sensitivity

Contrast sensitivity is a measure of the ability to discern different levels of luminance in a static image. It can be measured using Lea's contrast test or a Pelli Robson chart. Loss of contrast sensitivity caused by other ocular conditions will also affect the VA. People with low vision typically face difficulties in viewing objects or prints that have low contrast. Such persons are advised to adopt settings that offer good contrast, so that seeing becomes more comfortable, for example, rice in a dark bowl.

## Glare sensitivity

Is a measure of how quickly drivers can resume control of their vehicles when suddenly blinded by the lights of a vehicle approaching in the opposite direction. The glare is the result of excessive brightness within the visual field, due to various factors, such as cataract, corneal scarring, albinism, and retinal dystrophies. The glare can significantly reduce the VA. A brightness acuity tester (BAT) can be used to ascertain the visual disability caused due to glare. It can also be assessed by the reduction in visual acuity, following prolonged exposure to a light source.

## State of binocularity and stereopsis

The Worth's Four Dot Test, is used to diagnose the sensorial relationship between the two eyes. Red-green goggles dissociate the images seen by the two eyes. The testing is done at 20 feet and 13 inches, with the Worth-four-dot flashlight for macular fusion placed at a distance, and the peripheral fusion positioned in the proximity. This is a common adaptation to strabismus, amblyopia, and aniseikonia.

Stereopsis refers to the perception of depth or the relative proximity of an object. Determining the exact relationship of vehicles, animals and pedestrians, in the driving environment, is the most challenging aspect of the visual factors. It should be kept in mind that stereo acuity is not equivalent to depth perception, but is one of the many visual cues meant for determining the depth. This is tested using TNO and random-dot stereograms.

## Night vision

Defective night vision is seen in patients with retinal disorders, such as retinitis pigmentosa, and those with cataracts. It is also seen in people with intraocular lenses or those who underwent a refractive operation when a wide pupil exposed the edges of the IOL or of the ablation zone. The latter group of patients may also experience glare and loss of contrast sensitivity.

It is important for eye health workers to understand the functional assessment of vision, as it directly translates into visual efficiency in daily activities. This will not only aid in the development and interpretation of policies but also help in designing an enabling an ideal environment for people with visual impairment or any other disabilities.

